# Mucosal-Associated Invariant T Cells in the Human Gastric Mucosa and Blood: Role in *Helicobacter pylori* Infection

**DOI:** 10.3389/fimmu.2015.00466

**Published:** 2015-09-17

**Authors:** Jayaum S. Booth, Rosangela Salerno-Goncalves, Thomas G. Blanchard, Seema A. Patil, Howard A. Kader, Anca M. Safta, Lindsay M. Morningstar, Steven J. Czinn, Bruce D. Greenwald, Marcelo B. Sztein

**Affiliations:** ^1^Center for Vaccine Development, University of Maryland School of Medicine, Baltimore, MD, USA; ^2^Department of Pediatrics, University of Maryland School of Medicine, Baltimore, MD, USA; ^3^Department of Medicine, University of Maryland School of Medicine, Baltimore, MD, USA; ^4^Division of Gastroenterology and Hepatology, University of Maryland School of Medicine, Baltimore, MD, USA

**Keywords:** gastric MAIT, stomach, cytotoxic, *H. pylori*, age-related

## Abstract

Mucosal-associated invariant T (MAIT) cells represent a class of antimicrobial innate-like T cells that have been characterized in human blood, liver, lungs, and intestine. Here, we investigated, for the first time, the presence of MAIT cells in the stomach of children, adults, and the elderly undergoing routine endoscopy and assessed their reactivity to *Helicobacter pylori* (*H. pylori – Hp*), a major gastric pathogen. We observed that MAIT cells are present in the lamina propria compartment of the stomach and display a similar memory phenotype to blood MAIT cells. We then demonstrated that gastric and blood MAIT cells are able to recognize *H. pylori*. We found that CD8^+^ and CD4^−^CD8^−^ (double negative) MAIT cell subsets respond to *H. pylori*-infected macrophages stimulation in a MR-1 restrictive manner by producing cytokines (IFN-γ, TNF-α, IL-17A) and exhibiting cytotoxic activity. Interestingly, we observed that blood MAIT cell frequency in Hp^+ve^ individuals was significantly lower than in Hp^−ve^ individuals. However, gastric MAIT cell frequency was not significantly different between Hp^+ve^ and Hp^−ve^ individuals, demonstrating a dichotomy between blood and gastric tissues. Further, we observed that the majority of gastric MAIT cells (>80%) expressed tissue-resident markers (CD69^+^ CD103^+^), which were only marginally present on PBMC MAIT cells (<3%), suggesting that gastric MAIT cells are readily available to respond quickly to pathogens. These results contribute important new information to the understanding of MAIT cells function on peripheral and mucosal tissues and its possible implications in the host response to *H. pylori*.

## Introduction

Innate T cells are distinct from conventional T cells in that they have a limited repertoire diversity and that their responses display innate-like properties (1). Three subsets of innate T cells have been implicated in detection and response to pathogens, i.e., natural killer T cells (NKT), gamma-delta (γδ) T cells, and mucosal-associated invariant T (MAIT) cells (2–4). These cell subsets are phylogenetically conserved, express defined chemokine receptors, and are localized in peripheral tissues, particularly in mucosal tissues (5, 6). While NKT and γδ T cells have been studied extensively, much less information is available regarding MAIT cells characterization and function. In humans, intestinal innate T cells have been described in two distinct compartments: the lamina propria (LP) and the epithelial layer (5, 7). Although NKT and γδ T cells have been characterized in the gastric mucosa (8, 9), the presence of MAIT cells has not been reported. We have previously shown that gastric LP mononuclear cells (LPMCs) obtained from healthy volunteers contained more CD8^+^ T cells than CD4^+^ T cells, making the presence of CD8^+^ MAIT cells in the gastric milieu a distinct possibility (10).

Mucosal-associated invariant T cells are unique MHC-Ib-restricted T lymphocytes that express a semi-invariant T-cell receptor (Vα7.2 in humans) together with high levels of the NK receptor CD161. While the majority of MAIT cells display a CD8^+^ (αα or αβ) phenotype, double-negative (DN; CD4^−^CD8^−^) and CD4^+^ MAIT cells have also been reported (11). MAIT cells have been described as effector memory cells (5, 12). Recent studies have shown that human MAIT cells have the capacity to react rapidly to pathogens by producing IFN-γ and TNF-α in response to antigen-presenting cells (APCs) infected with several organisms, including *Mycobacterium tuberculosis*, *Salmonella enterica* serovar Typhimurium, and *Salmonella typhi* (4, 13, 14). MAIT cells do not, however, react to all pathogens (4). For example, MAIT cells were unable to respond to either *Listeria monocytogenes* or *Streptococcus* (4).

*Helicobacter pylori* (*H. pylori*) is a Gram-negative, microaerophilic bacterium that colonizes the human stomach, establishing a chronic infection in about two-thirds of the World population (15). *H. pylori* infection increases the risk for several clinical disorders such as gastritis, peptic ulcer, and gastric adenocarcinoma (15). The present investigations were undertaken to determine whether MAIT cells, if present in the gastric mucosa, might play a role in *H. pylori* infection. The presence of the riboflavin synthesis pathway in *H. pylori* supports the notion that these bacteria could produce the ligands required for stimulation of MAIT cells.

In this study, we showed for the first time that MAIT cells are present in the human gastric mucosa and display a memory phenotype similar to that observed in blood. Furthermore, we demonstrated that CD8^+^ and DN MAIT subsets are activated, in an MR-1-restricted manner, by *H. pylori*-infected macrophages (Mϕ) as evidenced by a robust production of cytokines and cytotoxic ability to lyse *H. pylori*-infected macrophages. We also observed significantly lower frequencies of CD8^+^ MAIT cells in blood, but not in the gastric mucosa, of Hp^+ve^ volunteers when compared with Hp^−ve^ volunteers. This is the first demonstration of MAIT cell subsets in the human stomach and their response to *H. pylori* infection, significantly extending our understanding of the role of MAIT cells in peripheral and mucosal tissues.

## Materials and Methods

### Volunteers

Volunteers were recruited from the Baltimore–Washington metropolitan area and University of Maryland, Baltimore, campus. Written informed consent was obtained from volunteers, and all procedures were approved by the University of Maryland, Baltimore Institutional Review Board. Blood and gastric biopsies were collected from 46 clinically indicated esophagogastroduodenoscopy (EGD) volunteers [children: 7–17 years (*n* = 12); adult: 18–64 years (*n* = 24); elderly: 65–85 years (*n* = 10)]. The indications for EGD included abdominal pain, heartburn, GERD, dysphagia, and acute gastritis. Diagnostic pathology reports showed that the stomach’s antral mucosa for the volunteers was either normal or exhibited mild inflammation. No concurrent GI diseases/disorders or other illnesses that may affect the GI tract were present. The presence of *H. pylori* infection was evaluated by culture and rapid urease test (CLO test) (16). All volunteers were *H. pylori* negative except where indicated in the narrative. In addition, PBMC collected from 11 healthy adult volunteers were also used in this study. PBMCs were isolated immediately after blood draws by density gradient centrifugation and cryopreserved in liquid nitrogen following standard techniques (17).

### Isolation of LPMCs from gastric biopsies

Gastric LPMCs were isolated as described previously (10). Briefly, after collection of biopsies from clinically indicated EGD volunteers, tissues were treated with HBSS (without CaCl_2_, MgCl_2_, MgSO_4_) (Gibco, Carlsbad, CA, USA) and EDTA (1 mM; Ambion, Grand Island, NY, USA) to remove intraepithelial cells. LPMCs were then isolated following enzymatic digestion of the biopsies with collagenase D (100 μg/ml; Roche, Indianapolis, IN, USA) and DNase I (10 μg/ml; Affymetrix, Cleveland, OH, USA) and homogenization using the Bullet Blender homogenizer (Next Advance Inc., Averill, NY, USA). Cells were then washed and resuspended in complete medium [RPMI 1640 (Gibco Invitrogen, Carlsbad, CA, USA) supplemented with 10% heat-inactivated fetal bovine serum (BioWhittaker, Walkersville, MD, USA), 2 mM l-glutamine (HyClone, Logan, UT, USA), 2.5 mM sodium pyruvate (Gibco), and 10 mM HEPES (Gibco), 100 U/ml penicillin (Sigma-Aldrich, St. Louis, MO, USA), 100 μg/ml streptomycin (Sigma-Aldrich), and 50 μg/ml gentamicin (Gibco)] and counted using Kova Glastic Slides (Hycor Biomedical, CA, USA). Cells were either stained immediately for immunophenotyping by flow cytometry or overnight stimulated with mitogens before staining (see below).

### *H. pylori* growth conditions

*H. pylori* strain 26695 (ATCC, Manassas, VA, USA) was grown on Columbia blood agar (Difco) containing 7% defibrinated horse blood (Hemostat Laboratories, Dixon, CA, USA), amphotericin B (2.5 μg/ml), and the selective antibiotics trimethoprim (20 μg/ml), vancomycin (6 μg/ml), and cefsulodin (16 μg/ml) (Sigma-Aldrich). Cultures were grown in a designated CO_2_ incubator with a humidity tray at 37°C and 10% CO_2_ for 72–96 h. In preparation for coculture assays with THP-1 macrophages, bacteria were transferred to 10 ml Brucella broth (Difco) containing 10% FBS plus antibiotics in 25-cm^2^ tissue culture flasks overnight. Bacterial density was determined by obtaining readings at an optical density of 450 nm (OD, 450) and comparing them to a standardized growth curve, a value of 0.071 corresponding to 1 × 10^7^ bacteria/ml.

### Preparation of *H. pylori* lysate antigen

*H. pylori* strain 26695 was grown on Columbia agar (Difco) supplemented with 7% horse blood under microaerobic conditions (5% O_2_, 10% CO_2_) at 37°C. After 96 h, bacteria were harvested and cultured in tissue culture flasks containing Brucella broth (Difco) supplemented with 10% fetal bovine serum. Cultures were grown at 37°C with 5% CO_2_. Bacterial cultures were recovered by centrifugation at 4,000 × *g* for 20 min and then suspended in 2 ml phosphate-buffered saline (PBS). Bacteria were lysed by 4 × 60 s bursts of power using a probe sonicator (Sonics and Materials Inc., Danbury, CT, USA). Whole bacteria were removed by centrifugation at 5,000 × *g* for 20 min and passing the supernatant through a 0.22-μm pore filter (18).

### Culture, differentiation, and infection of THP-1

The human monocyte cell line THP-1 (ATCC catalog # TIB-202) was cultured and differentiated as described previously (19). Briefly, THP-1 cells were cultured in complete RPMI described above at 5% CO_2_ at 37°C. THP-1 cells were then differentiated into macrophages (Mϕ) by incubating with phorbol 12-myristate 13-acetate (PMA) (50 ng/ml; Sigma-Aldrich) for 48 h at 37°C in 5% CO_2_. The level of cell differentiation was evaluated by surface staining of the cells with antibodies against the classical macrophage marker CD68 (Y1/82A, Biolegend, San Diego, CA, USA). Cells were then washed and incubated overnight in complete RPMI before infection with *H. pylori*. THP-1-differentiated Mϕ were washed in antibiotics-free medium and counted using the viability dye Trypan blue to determine the number of viable THP-1 macrophages. A total of 1 × 10^6^ THP-1 were incubated in complete RPMI without antibiotics for 4 h at 37°C in 5% CO_2_ in the absence or presence of *H. pylori* at 5, 10, 20, 50, and 100 multiplicity of infection (MOI). The infection was stopped by the addition of fresh complete RPMI 1640 medium containing gentamicin (100 μg/ml; Sigma-Aldrich). Cells were then labeled with CD45, a marker present in all hematopoietic cells, to exclude infected Mϕ from flow cytometry analysis. To allow the cells to recover from the infection, infected Mϕ were rested for overnight at 37°C before coculturing with PBMC. Negative controls (i.e., non-infected Mϕ) were treated identically except that they were not infected with *H. pylori*. The levels of infection were determined both by flow cytometry using a goat-anti-*Helicobacter pylori* antiserum (02-03-94, KPL, Gaithersburg, MD, USA) and by bacteria entry assays as described below.

### Bacteria entry assay

Bacteria entry experiments were performed as previously described (20). Briefly, after gentamicin treatment, 2 × 10^5^ Mϕ were lysed using 0.1% saponin in PBS. Lysates were plated in serial dilution on solid GC agar plates supplemented with 10% horse serum and vancomycin for 48 h under microaerobic conditions, 10% CO_2_ at 37°C to detect intracellular bacteria.

### Isolation and culture of primary macrophages

Primary macrophages cells were isolated from PBMC by adhesion to tissue culture plastic plates as described previously (21). Macrophages were >90% pure as determined by staining with CD14 and CD68. Isolated primary macrophages were harvested using a rubber policeman and were then infected with *H. pylori* at MOI of 20. For blocking experiments, *H. pylori*-infected macrophages were pre-treated with anti-MR-1 mAb (10 μg/ml, clone 26.5, Biolegend) or their matched isotype control mouse IgG2a (10 μg/ml, MOPC-173, Biolegend) before coculture with autologous effector cells.

### Stimulation of PBMC and gastric LPMC for flow cytometry analyses

*Ex vivo* PBMCs were used as effector cells. Briefly, PBMCs were cocultured with live *H. pylori* (MOI of 50), *H. pylori* lysate (homogenate; 5 μg/ml), non-infected Mϕ, or *H. pylori*-infected Mϕ (MOI of 20). The effector:*H. pylori*-infected Mϕ ratios were 50:1, 20:1, 10:1, and 5:1. PBMCs cultured with media only or in the presence of staphylococcal enterotoxin B (SEB) (10 μg/ml; Sigma) were used as negative and positive controls, respectively. Freshly isolated gastric LPMCs were cocultured with media alone, non-infected Mϕ, *H. pylori*-infected Mϕ (MOI of 20) (Effector:*H. pylori* infected-Mϕ ratio was 50:1), or anti-CD3/CD28 beads (positive control; Life technologies, Grand Island, NY, USA). In some experiments (see Results), at the time of stimulation, anti-human CD107a-FITC (5 μl; H4A3, BD, San Jose, CA, USA) was added. After 4 h, 0.5 μl of Golgi Stop (Monensin, BD, USA) and Golgi Plug (Brefeldin A, BD, USA) were added and cultures continued overnight at 37°C in 5% CO_2_.

### Surface and intracellular staining

Following stimulation, PBMCs and LPMCs were stained for flow cytometry analysis as previously described (10). Following stimulation in the presence of CD107a [LAMP-1, a molecule expressed on the cell membrane which is widely accepted to be associated with cytotoxic T-cell activity (22)), PBMCs and LPMCs were stained for live/dead discrimination (YEVID) (Invitrogen, Carlsbad, CA, USA). Blocking of Fc receptors was performed using human immunoglobulin (3 μg/ml; Sigma) and was followed by surface staining. Briefly, cells were stained with fluorescently labeled monoclonal antibodies (mAbs) directed to CD14-BV570 (M5E2, Biolegend, San Diego, CA, USA), CD19-BV570 (HIB19, Biolegend), CD3-BV650 (OKT3, Biolegend), CD4-PerCP-Cy5.5 (SK3, BD), CD8-biotin (RPA-T8, BD), CD45RA-BV605 (HI100, Biolegend), TCR Vα7.2-PE (3C10, Biolegend), CD103-Alexa-Fluor 488 (Ber-ACT8, Biolegend), CD161-APC (DX12, BD), and CD62L-Alexa Fluor 780 (DREG-5, eBioscience, San Diego, CA, USA) at 4°C for 30 min. Staining with streptavidin-QDot800 (Invitrogen) was performed for panels that included biotin-conjugated mAbs for 30 min at 4°C. Cells were then fixed and permeabilized using IC fixation and permeabilization buffers (8222/8333, eBioscience) according to the manufacturer’s recommendations. This was followed by intracellular staining with mAbs directed to IL-17A-BV421 (BL168, Biolegend), IFNγ-PE-Cy7 (B27, BD), TNFα-Alexa 700 (MAb11, BD), and CD69-ECD (TP1.55.3, Beckman Coulter, Danvers, MA, USA) which was performed at 4°C overnight. After staining, cells were stored in 1% paraformaldehyde at 4°C until data collection. Data were collected using a customized LSRII flow cytometer (BD) and then analyzed using the WinList version 7 (Verity Software House, Topsham, ME, USA) software package. Functional responses were considered specific for *H. pylori*-infected autologous macrophages if the differential in the number of positive and negative events between experimental (*H. pylori*-infected Mϕ) and negative control (uninfected Mϕ) cultures were significantly increased (*P* < 0.05) using *z*-tests.

### Cytotoxic T lymphocyte assays

*In vitro*-expanded PBMC from volunteers were obtained and used for cytotoxic T lymphocyte (CTL) as previously described for *S. typhi* (17). Briefly, PBMCs were cocultured with stimulator cells at an effector to stimulator cell ratio of 7:1 in complete RPMI supplemented with 20 IU/ml of rhIL-2 for 7–8 days. Stimulator cells consisted of THP-1 Mϕ infected with *H. pylori* (MOI-20). After infection, THP-1 Mϕ were rested overnight and gamma-irradiated (4,000 rads). For blocking experiments, stimulators were pre-treated for 1 h with anti-MR1 polyclonal antibodies [10 μg/ml, Santa Cruz Biotechnology (SC), San Diego, CA, USA], or anti-MR1 mAb (10 μg/ml, clone 26.5, kindly provided by Dr. Ted H. Hansen) or their matched controls (10 μg/ml), goat IgG (Gene Tex Inc, Irvine, CA, USA) and mouse IgG2a (MOPC-173, Biolegend), respectively, before coculture with effector cells. Cytotoxicity was determined by a 4-h ^51^Cr-release assay as previously described (17). Briefly, serial twofold dilutions of expanded effector cells (1.87 × 10^4^ to 1.5 × 10^5^ cells/well) were incubated with *H. pylori*-infected and non-infected Mϕ target cells (3 × 10^3^ cells/well) previously labeled with 200 μCi of ^51^CrO_4_Na_2_ (MP Biomedical, Solon, OH, USA). Cultures were centrifuged at 50 × *g* for 5 min and incubated for 4 h at 37°C in the presence of 5% CO_2_. Supernatants were then collected and transferred to 96-well plates containing scintillation liquid (OptiPhase; LKB Wallac, Gaithersburg, MD, USA). The amount of ^51^Cr released was measured with a Wallac Trilux β-counter (LKB Wallac). Cultures were tested in triplicate wells. The percentage of cytotoxicity was calculated as follows: (experimental release − spontaneous release)/(maximal release − spontaneous release) × 100, where spontaneous release is the counts per minute released by target cells in the absence of effector cells and maximal release is the counts per minute released in the presence of 5% Triton X-100 as previously described (17).

### Statistical analysis

Data were analyzed using the statistical software GraphPad Prism™ version 5.03 (Graphpad, San Diego, CA, USA). Kruskal–Wallis ANOVA and Dunn’s post-test were used to determine significant differences between multiple groups and to compare selected group pairs. Statistical differences in median values between two groups were determined using Mann–Whitney tests. Correlations between MAIT cell frequencies and age were evaluated using Spearman’s tests.

## Results

### MAIT cells are present in the stomach

**Figure 1:**
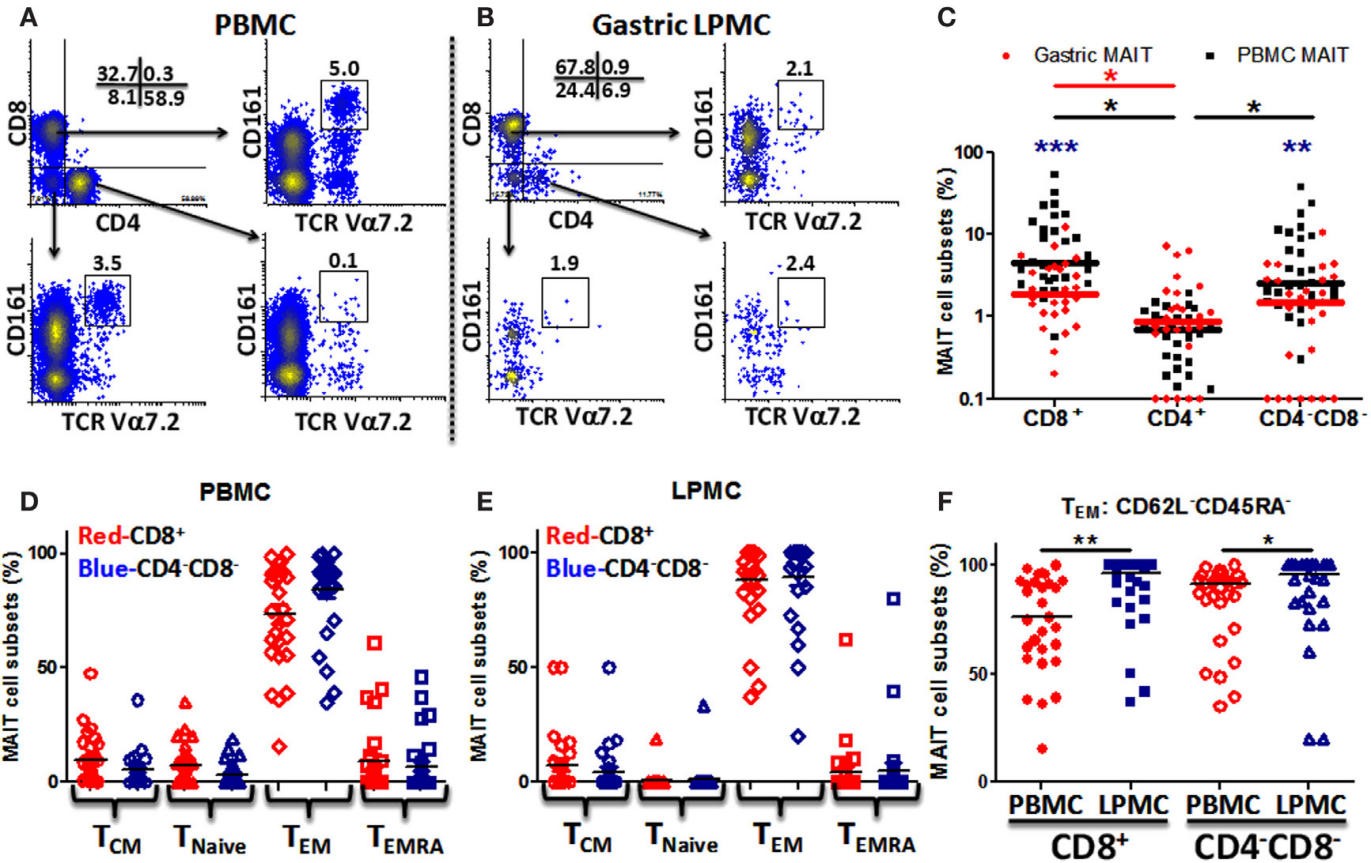
**MAIT cells are present in the human gastric mucosa and exhibit T effector memory phenotype**. **(A)** Identification of MAIT cell subsets [CD8^+^, CD4^+^, and CD4^−^CD8^−^ (DN)] in PBMC and **(B)** in gastric LPMC in a representative individual as CD161^hi^ TCR Vα7.2 (box). **(C)** Comparison of gastric (red dots; *n* = 27) and PBMC (black squares; *n* = 29) MAIT cell subsets. Significant differences between MAIT cell subsets in (i) PBMC [black asterisks (*)] and (ii) gastric LPMC [red asterisk (*)]. Blue asterisks (*) denote significant differences in MAIT cell subsets between PBMC and LPMC. Horizontal lines represent medians (red bars: gastric MAIT; black bars: PBMC MAIT). **(D)** Expression of CD45RA and CD62L to evaluate memory [T_CM_ (CD62L^+^CD45RA^−^), T_EM_ (CD62L^−^CD45RA^−^), and T_EMRA_ (CD62L^−^CD45RA^+^)] and T_naive_ (CD62L^+^CD45RA^+^) subpopulations in MAIT subsets from PBMC (*n* = 29) and **(E)** LPMC (*n* = 27). **(F)** Comparison of T_EM_ MAIT cell subsets between PBMC and LPMC. *P < 0.05; **P < 0.005; ***P < 0.0005.

To evaluate whether MAIT cells are present in the human stomach, we isolated LPMCs from gastric biopsies obtained from various age groups and characterized them by flow cytometry. We then compared the phenotype and function of MAIT cells present in the stomach with those present in the blood. As expected, blood MAIT cells were detected in the three major T-cell subsets CD8^+^, CD4^+^, and CD4^−^CD8^−^ (DN) (Figure 1A; Figure S1 in Supplementary Material). Although at different proportions, gastric MAIT cells were also composed of three different subsets: CD8^+^, CD4^+^, and DN MAIT cell subsets (Figure 1B; Figure S1 in Supplementary Material). Cumulative data showed that the percentages of gastric CD8^+^ and DN MAIT cell subsets were significantly lower than their counterpart MAIT subsets in blood (Figure 1C). In addition, the percentages of blood and gastric CD8^+^ and DN MAIT cell subsets were significantly higher than CD4^+^ MAIT subsets (Figure 1C).

Since MAIT cells have been shown to display a T-cell effector memory (T_EM_) phenotype (5, 12), we evaluated the phenotype of blood and gastric MAIT cell subsets by assessing CD45RA and CD62L expression in the two major MAIT cell subsets (CD8^+^ and DN). Blood MAIT cell subsets showed comparable levels of T_EM_ (CD62L^−^CD45RA^−^) in CD8^+^ and DN MAIT subsets (Figure 1D). Similarly, phenotypic analysis of gastric MAIT cell subsets showed that CD8^+^ and DN MAIT subsets displayed similar levels of T_EM_ phenotypes (~70–80%) (Figure 1E). We then compared blood and gastric MAIT cell subtypes displaying the T_EM_ phenotype. We found that gastric LPMCs have significantly higher MAIT cells expressing a T_EM_ phenotype than blood in both MAIT subsets (Figure 1F).

**Figure 2:**
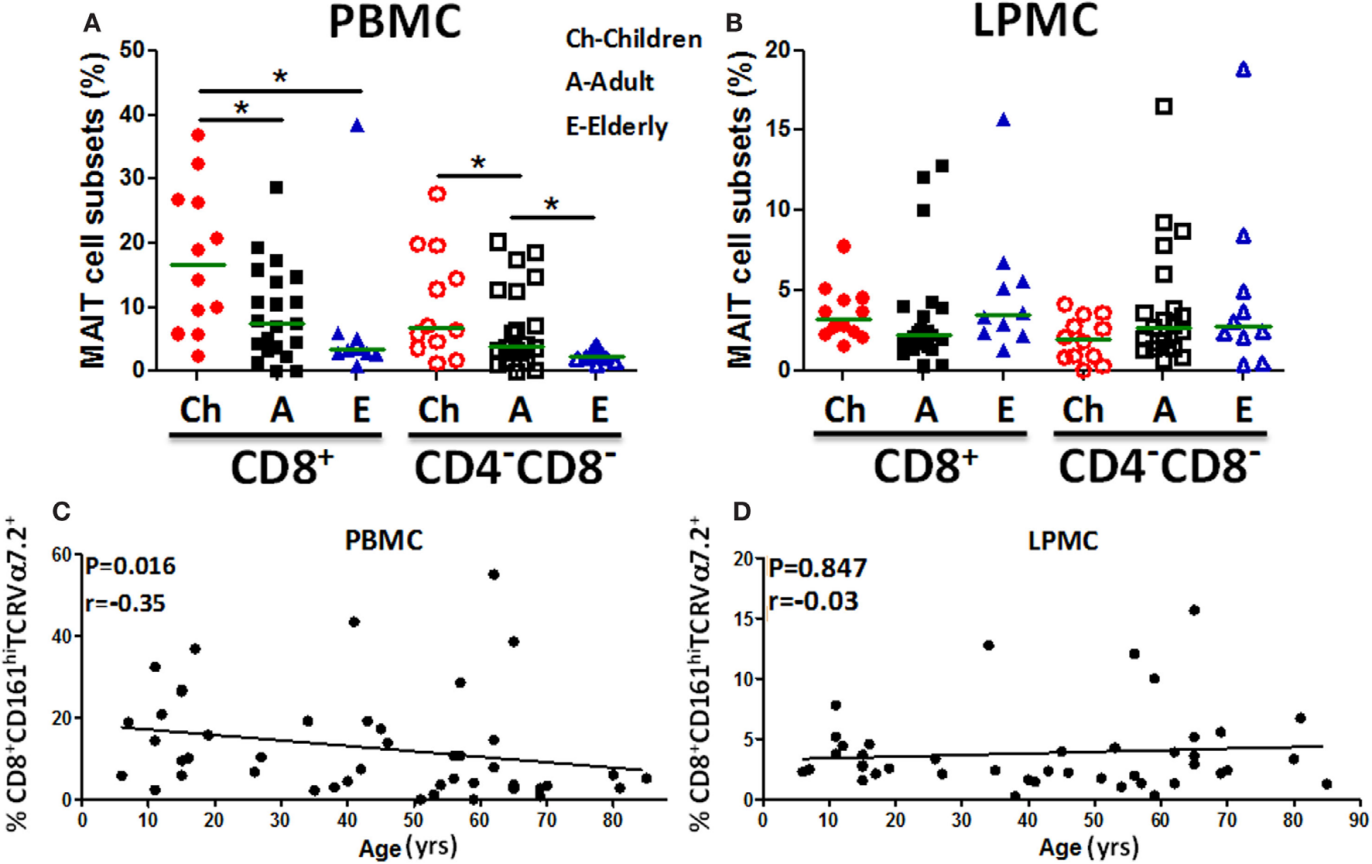
**MAIT cells in blood (PBMC) and gastric LPMC in children, adults, and the elderly**. **(A)** PBMC and **(B)** gastric LPMC obtained from children (*n* = 12), adults (*n* = 21), and the elderly (*n* = 10) were evaluated for the presence of MAIT cells. Significant differences among age groups are denoted by asterisks (*P < 0.05). **(C)** Correlation of CD8^+^ MAIT cells with age in PBMC (*n* = 46) and **(D)** in gastric LPMC (*n* = 43) was performed using Spearman’s correlation analysis. Horizontal green lines in **(A,B)** represent medians.

Because the presence of MAIT cells in the human gastric mucosa was not previously reported, we determined their frequency in gastric biopsies obtained from children, adults, and the elderly and compared these frequencies to blood MAIT cells. The frequencies of both MAIT cell subsets (CD8^+^ and DN) in blood were found to be significantly higher in children than in adults and the elderly (Figure 2A). In contrast, in LPMC, although some trends were noted, MAIT cell subsets frequencies were not different among the three age groups (Figure 2B). Using Pearson’s regression analysis, we observed a significant inverse correlation (*r* = −0.35, *P* = 0.016) between the frequency of the CD8^+^ MAIT subset and age in PBMC but not in LPMC (*r* = −0.03, *P* = 0.847) (Figures 2C,D). Regression analysis of DN subsets showed similar results, i.e., significant inverse correlations between the frequencies of blood DN (*r* = −0.33, *P* = 0.022) MAIT subsets and age (Figure S2A in Supplementary Material). Similar to LPMC CD8^+^ MAIT, DN MAIT subset showed a trend to be correlated (*r* = 0.25, *P* = 0.10) with age (Figure S2B in Supplementary Material).

### Activation of blood MAIT cells with live *H. pylori* or lysate

The observation that MAIT cells were present at the gastric mucosa led us to speculate that MAIT cells might play a role in *H. pylori* infection, a major gastric pathogen. To explore this possibility, we determined the ability of blood MAIT cells from individuals to be activated following exposure to *H. pylori* antigens or live *H. pylori* bacteria by assessing their cytokines/cytotoxic (IFN-γ, TNF-α, and CD107a) responses. Blood MAIT cells were characterized using the gating strategy described in Figure 1A and Figure S1 in Supplementary Material. While exposure to *H. pylori* antigens (lysate) did not stimulate blood CD8^+^ MAIT cells, exposure of these cells to live *H. pylori* bacteria (MOI of 50) resulted in increased expression of CD107a (5.3%) and low levels of TNF-α production (0.8%) (Figure S3A in Supplementary Material). As expected, the positive control, SEB, stimulated CD8^+^ MAIT cells to produce high levels of IFN-γ and TNF-α and up-regulate CD107a expression, demonstrating that these cells were functional (Figure S3A in Supplementary Material).

### Blood CD8^+^ MAIT cells activation by *H. pylori*-infected macrophages

Given the modest response of MAIT cells to live *H. pylori* stimulation, we explored whether stimulation of blood CD8^+^ MAIT cells with *H. pylori-*infected macrophages (Mϕ) resulted in increased levels of cell activation. This possibility was supported by previous reports showing that macrophages, B cells, and epithelial cells infected with microbes resulted in higher levels of MAIT cell stimulation (5, 14, 23). We first optimized the preparation of *H. pylori-*infected targets by infecting THP-1 Mϕ with *H. pylori* at different MOI (10, 20, 50, and 100) and evaluating the infection rates in differentiated (CD68^+^) vs. undifferentiated (CD68^−^) Mϕ using an anti-*H. pylori* antibody and flow cytometry (Figures S3B,C in Supplementary Material). We observed that *H. pylori* were able to infect THP-1 Mϕ and the majority of the infected cells were differentiated Mϕ (Figure S3B in Supplementary Material). Of note, although at 50 and 100 MOI the rates of infection showed the highest percentages of *H. pylori*^+^ cells, the viability of the Mϕ at these MOI decreased to about 70% (Figure S3C in Supplementary Material). To further confirm these results, *H. pylori*-infected Mϕ were lysed and plated on agar plates. In agreement with flow cytometric assays, a dose-dependence effect was observed where the infection level was proportional to the *H. pylori* MOI dose (data not shown). Based on these results, an MOI of 20:1 was chosen for subsequent experiments in the preparation of *H. pylori*-infected Mϕ.

**Figure 3:**
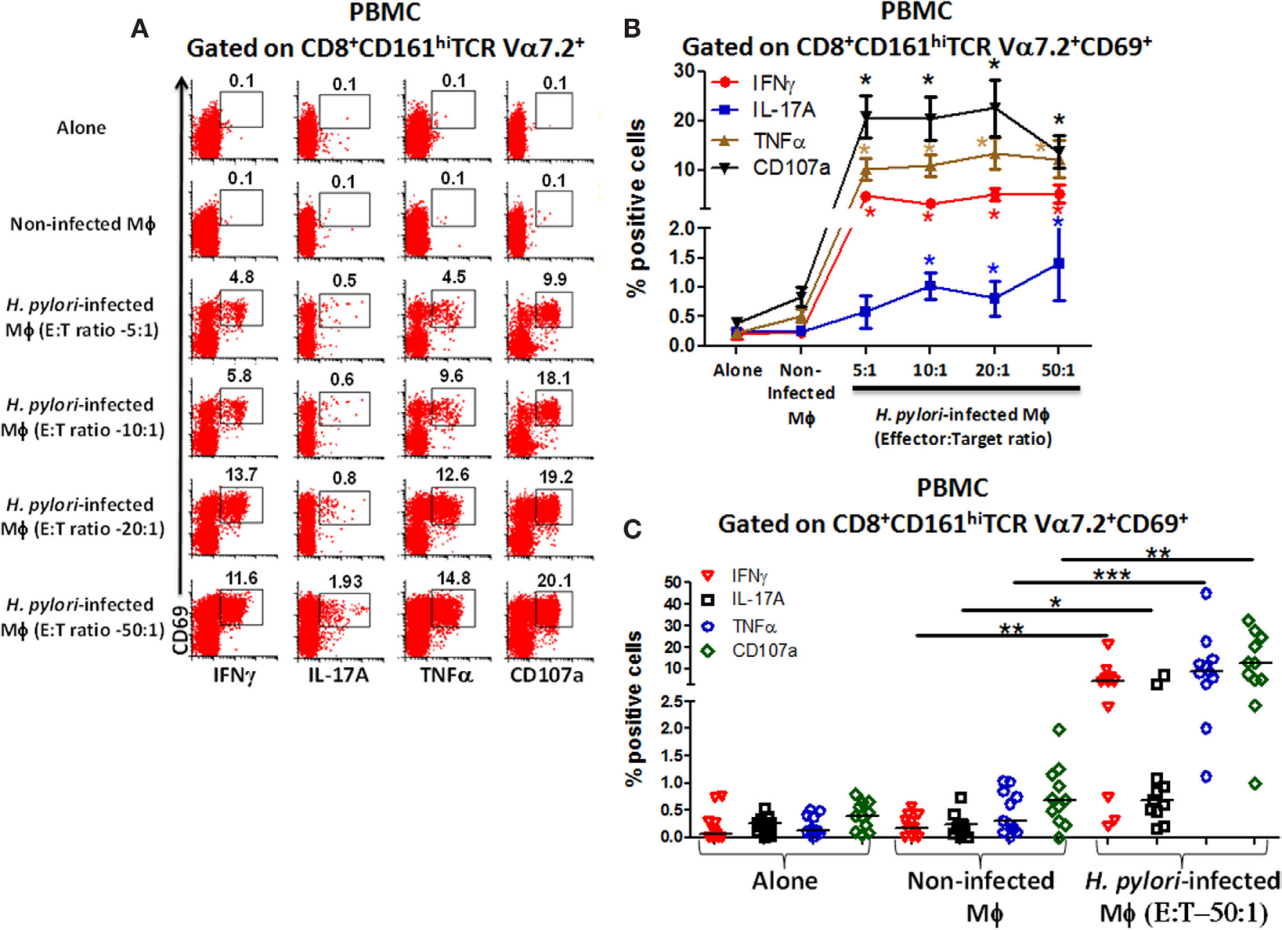
**Responses of blood CD8^+^ MAIT cells from healthy adults to *H. pylori*-infected macrophages**. **(A)** Representative volunteer showing the induction of cytokine production (IFN-γ, TNF-α, IL-17A) and up-regulation of CD107a expression by CD8^+^ MAIT cells following stimulation by *H. pylori-*infected differentiated THP-1 macrophages (Mϕ) at increasing effector to target (E:T) ratios (5:1, 10:1, 20:1, 50:1). **(B)** Cumulative data (*n* = 11) displaying the percentages of cytokine-producing and CD107a-expressing CD8^+^ MAIT cells following stimulation with *H. pylori*-infected Mϕ (E:T 5:1, 10:1, 20:1, 50:1), non-infected Mϕ, and effector cells alone. Shown are significant differences (*) between non-infected and infected targets. Significances were *P* < 0.0005 for CD107 and TNF-α and *P* < 0.005 for IFN-γ at all E:T ratios and *P* < 0.05 for IL-17A at 10:1, 20:1, and 50:1 E:T ratios. **(C)** Cumulative data (*n* = 11) showing cytokine production and expression of CD107a following stimulation with *H. pylori*-infected Mϕ at E:T (50:1) ratio, non-infected Mϕ, and effector cells alone. Horizontal black lines in **(C)** represent medians. Significant differences were determined between non-infected and *H. pylori*-infected Mϕ (*P < 0.05; **P < 0.005; ***P < 0.0005).

To investigate whether blood CD8^+^ MAIT cells could be efficiently stimulated by *H. pylori*-infected Mϕ, MAIT cells (effectors) were exposed to either non-infected Mϕ or *H. pylori*-infected Mϕ (targets) with increasing effector:target (E:T) ratios (5:1, 10:1, 20:1, and 50:1) and cytokine production and CD107a expression measured by flow cytometry. Following stimulation with *H. pylori*-infected THP-1 Mϕ, higher percentages of CD8^+^ MAIT cells producing cytokines (IFN-γ, IL-17A, and TNF-α) and up-regulating expression of CD107a were detected than stimulation with non-infected THP-1 Mϕ or effectors (Figure 3A). Cumulative data (*n* = 11) showed that significantly (*P* < 0.05) higher percentages of blood CD8^+^ MAIT cells produced cytokines (IFN-γ, IL-17A, and TNF-α) and expressed elevated levels of CD107a following stimulation with *H. pylori*-infected Mϕ than when exposed to non-infected Mϕ (Figures 3B,C).

### Blood CD4^−^CD8^−^ (DN) MAIT cells activation by *H. pylori*-infected macrophages

Given that blood CD8^+^ MAIT cells are reactive to *H. pylori* and that MAIT cells were observed in DN, we next investigated whether blood DN MAIT subsets were able to respond to *H. pylori*-infected Mϕ.

**Figure 4:**
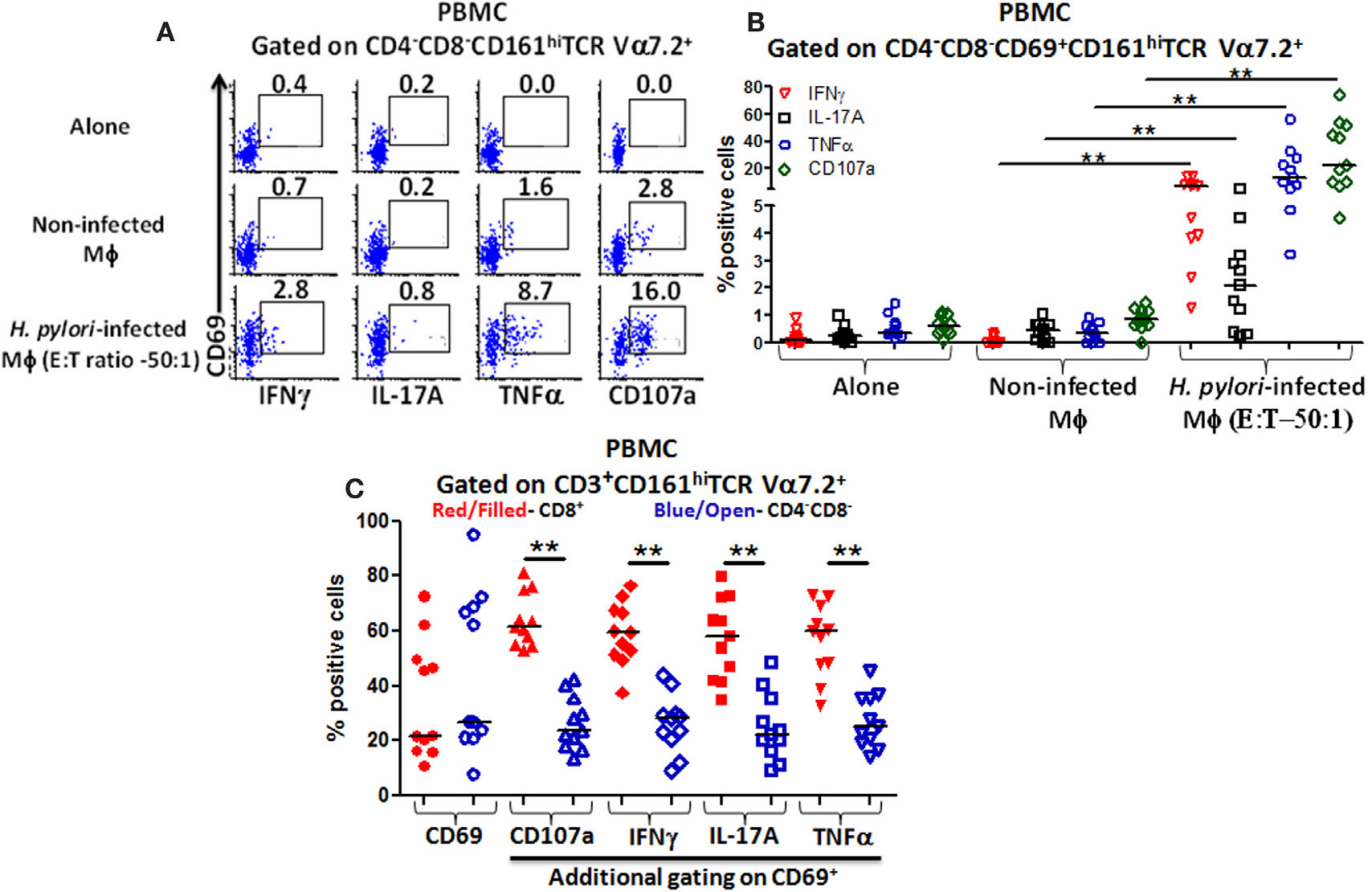
**Responses of blood CD4^−^CD8^−^ (DN) MAIT subsets from healthy adults to *H. pylori*-infected macrophages**. **(A)** Representative volunteer showing the induction of cytokine production (IFN-γ, TNF-α, IL-17A) and up-regulation of CD107a expression in DN MAIT cells following stimulation with media (alone), non-infected THP-1 macrophages (Mϕ), or *H. pylori*-infected THP-1 Mϕ (E:T – 50:1). **(B)** Cumulative data (*n* = 11) showing production of cytokines (IFN-γ, TNF-α, and IL-17A) and expression of CD107a by DN MAIT cells following stimulation with *H. pylori*-infected THP-1 Mϕ (E:T – 50:1). **(C)** CD69 up-regulation by CD3^+^CD161^+^TCR Vα7.2^+^ cells following *H. pylori*-infected THP-1 Mϕ stimulation (E:T – 50:1) and assessment of the level of cytokines production (IFN-γ, TNF-α, IL-17A) and CD107a expression in CD69^+^ MAIT cell subsets (CD8^+^ and CD4^−^CD8^−^) following stimulation with *H. pylori*-infected THP-1 Mϕ (E:T – 50:1) (*n* = 11). Horizontal black lines in **(B,C)** represent medians. Significant differences are denoted by asterisks (**P < 0.005).

We observed that higher percentages of DN MAIT cells produced cytokines (IFN-γ, TNF-α, and IL-17A) and up-regulated CD107a expression following stimulation with *H. pylori*-infected THP-1 Mϕ (E:T – 50:1) than when exposed to non-infected Mϕ or when effector cells were cultured alone (Figure 4A). Cumulative data (*n* = 11) showed that significantly higher percentages of DN MAIT cells produced cytokines (IFN-γ, IL-17, and TNF-α) and up-regulated CD107a following stimulation with *H. pylori*-infected Mϕ than with non-infected Mϕ (Figure 4B).

### Dominant subsets of blood MAIT cells following exposure to *H. pylori*-infected macrophages

Since both blood MAIT subsets (CD8^+^ and DN) were able to recognize and respond to *H. pylori*-infected THP-1 Mϕ, we then examined whether there were differences in the activation levels and quality of the responses between these subsets. Following stimulation with *H. pylori*-infected THP-1 Mϕ, increased expression of the activation marker CD69 was observed in both MAIT subsets compared with non-infected Mϕ (Figure 4C). Interestingly, both MAIT subsets exhibited similar percentages of CD69^+^ cells (Figure 4C), indicating that CD8^+^ and DN MAIT subsets are similarly activated by *H. pylori*-infected macrophages. However, further analysis of CD69^+^ MAIT cells indicated that although CD8^+^ and DN are equally activated (based on similar CD69^+^ expression levels), significantly higher percentages of CD8^+^ MAIT cells produced cytokines (IFN-γ, TNF-α, and IL-17A) and up-regulated CD107a than DN MAIT cells (Figure 4C). Thus, the cytokine responses and CD107a expression to *H. pylori*-infected THP-1 Mϕ are dominated by the CD8^+^ MAIT subset (>60%) (Figure 4C).

### Multifunctional MAIT cell responses following exposure to *H. pylori*-infected macrophages

**Figure 5:**
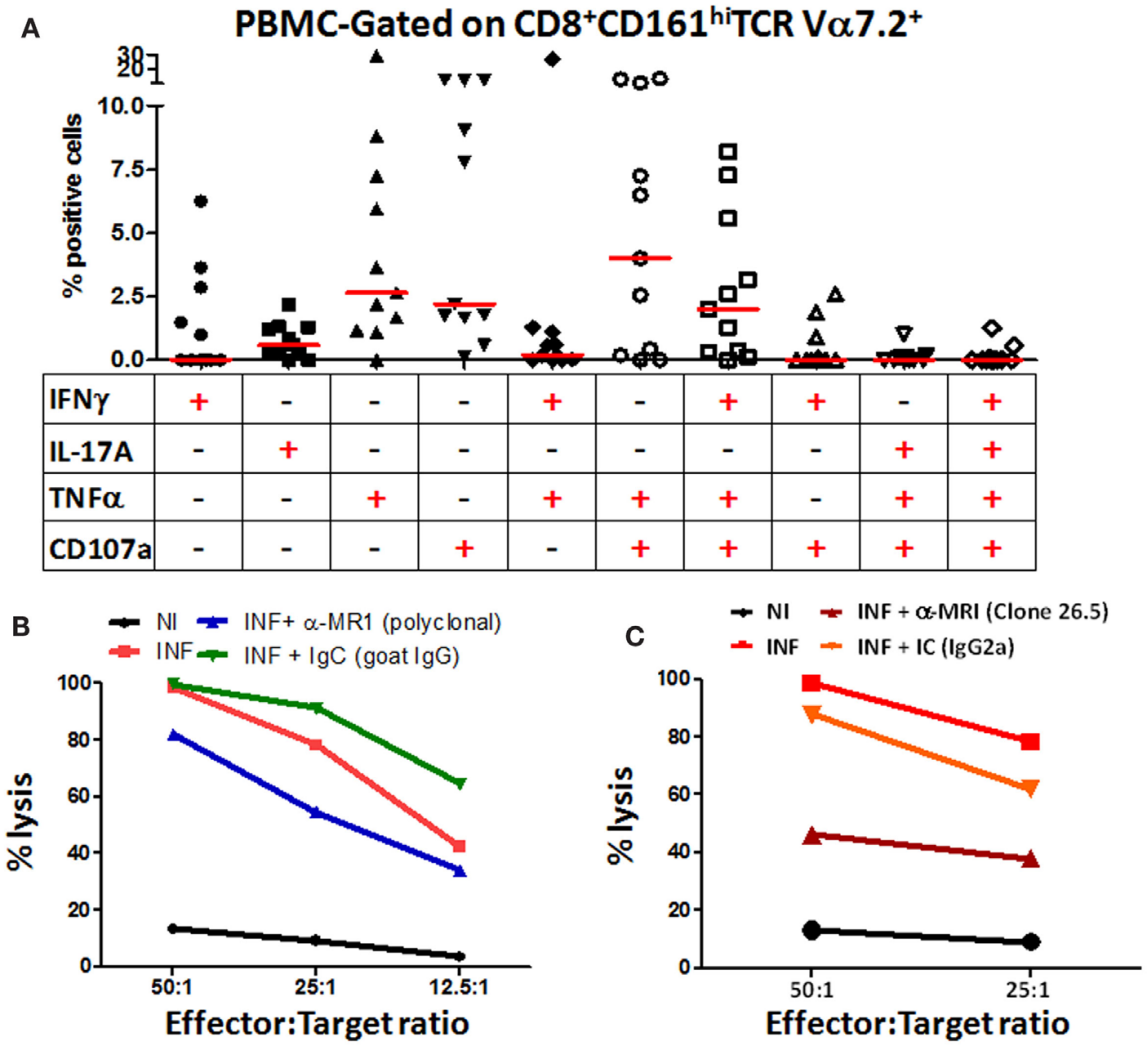
**Blood MAIT cells from healthy adults exhibit multifunctional abilities and are cytotoxic to *H. pylori*-infected macrophages**. **(A)** Multifunctional activities of MAIT cells were determined by simultaneous detection of three intracellular cytokines (IFN-γ, TNF-α, and IL-17A) and expression of CD107a by CD8^+^ MAIT cells following stimulation with *H. pylori-*infected THP-1 Mϕ (E:T – 50:1). Scatter plot shows single-cytokine-producing and CD107-expressing cells and the six predominant multi-cytokine-producing/CD107-expressing patterns using PBMCs from healthy adult volunteers. Horizontal red lines indicate the median responses. Multifunctionality was analyzed using the FCOM feature of WinList. **(B)** *H. pylori* CTL responses by MAIT cells were measured at various effector:target ratios (50:1, 25:1, 12.5:1, 6.25:1) in non-infected (NI) and *H. pylori*-infected (INF) THP-1 Mϕ. Blocking of CTL responses was performed using anti-human MR-1 antibody (goat, polyclonal) (10 μg/ml) or goat IgG control (immunoglobulin control; IgC; 10 μg/ml). **(C)** *H. pylori* CTL responses by MAIT cells were measured at two effector:target ratios (50:1 and 25:1) in NI and INF THP-1 Mϕ. Blocking of CTL responses were performed using the anti-human MR-1 mAb (clone 26.5) (10 μg/ml) or a matched isotype control (IC, 10 μg/ml). Lines show the mean percentages of cytotoxicity at different E/T ratios from triplicate wells. The data are representative of four separate experiments.

Our group has previously shown that MAIT cells can respond to *S. typhi* by secreting multiple cytokines simultaneously (14). Thus, we next investigated the multifunctionality of the responses exhibited by blood CD8^+^ MAIT cells against *H. pylori*-infected differentiated THP-1 macrophages. Analysis of multiple cytokines (IFN-γ, TNF-α, and IL-17A) and/or CD107 expression patterns (16 possible combinations) using the Winlist FCOM function revealed that MAIT cell responses were characterized by single, double, or triple cytokine producers/CD107 expressors, albeit at different percentages, following stimulation with *H. pylori*-infected THP-1 Mϕ (Figure 5A). Interestingly, CD8^+^ MAIT cells produced IL-17A mostly as single-cytokine-producing cell following stimulation with *H. pylori*-infected Mϕ. In contrast, CD8^+^ MAIT cells response to *H. pylori*-infected Mϕ produced IFN-γ mostly as multifunctional [triple-positive cells (IFN-γ^+^ TNF-α^+^ CD107a^+^)] (Figure 5A). We also noted that the cytotoxic marker, CD107a, was prominent in both multifunctional and single-positive cell subsets, suggesting that MAIT cells are highly cytotoxic to *H. pylori*-infected THP-1 Mϕ.

### MAIT cells are cytotoxic to *H. pylori*-infected macrophages

Because we observed a preponderance of the cytotoxic degranulation marker CD107a (LAMP-1) expressed on stimulated MAIT cells, we directly explored the functional cytotoxic ability of MAIT cells to kill *H. pylori*-infected targets using standard chromium (^51^Cr) release assays. We observed significant increases in target cell killing in cultures containing *H. pylori*-infected THP-1 Mϕ when compared to cultures with non-infected THP-1 Mϕ (Figure 5B). To assess whether this MAIT cell killing involved MR1-restriction, we treated the stimulator cells with anti-human MR1 antibodies or matched immunoglobulin or isotype controls to block the lysis of *H. pylori*-infected THP-1 Mϕ (Figures 5B,C). We observed that blocking of MR-1 using either a goat polyclonal (Figure 5B) or a mouse anti-human monoclonal (clone 26.5; Figure 5C) antibodies resulted in significant decreases in the lysis of *H. pylori*-infected THP-1 Mϕ when compared to cultures with *H. pylori*-infected targets (Figures 5B,C). However, the 26.5 mAb to MR1 was more effective in preventing lysis of *H. pylori* targets than the polyclonal goat MR1 antibody (Figures 5B,C). As expected, addition of matched immunoglobulin (IgC) or isotype (IC) controls failed to decrease the lysis of *H. pylori*-infected Mϕ observed in cultures exposed to *H. pylori*-infected targets (Figures 5B,C). These results confirmed that blood MAIT cells exhibit a cytotoxic effector function to *H. pylori*-infected THP-1 Mϕ cells and that this effect is MR-1 restricted.

### MAIT cell subsets (CD8^+^ and CD4^−^CD8^−^ DN) responses to *H. pylori*-infected primary autologous macrophages are MR-1 restricted

**Figure 6:**
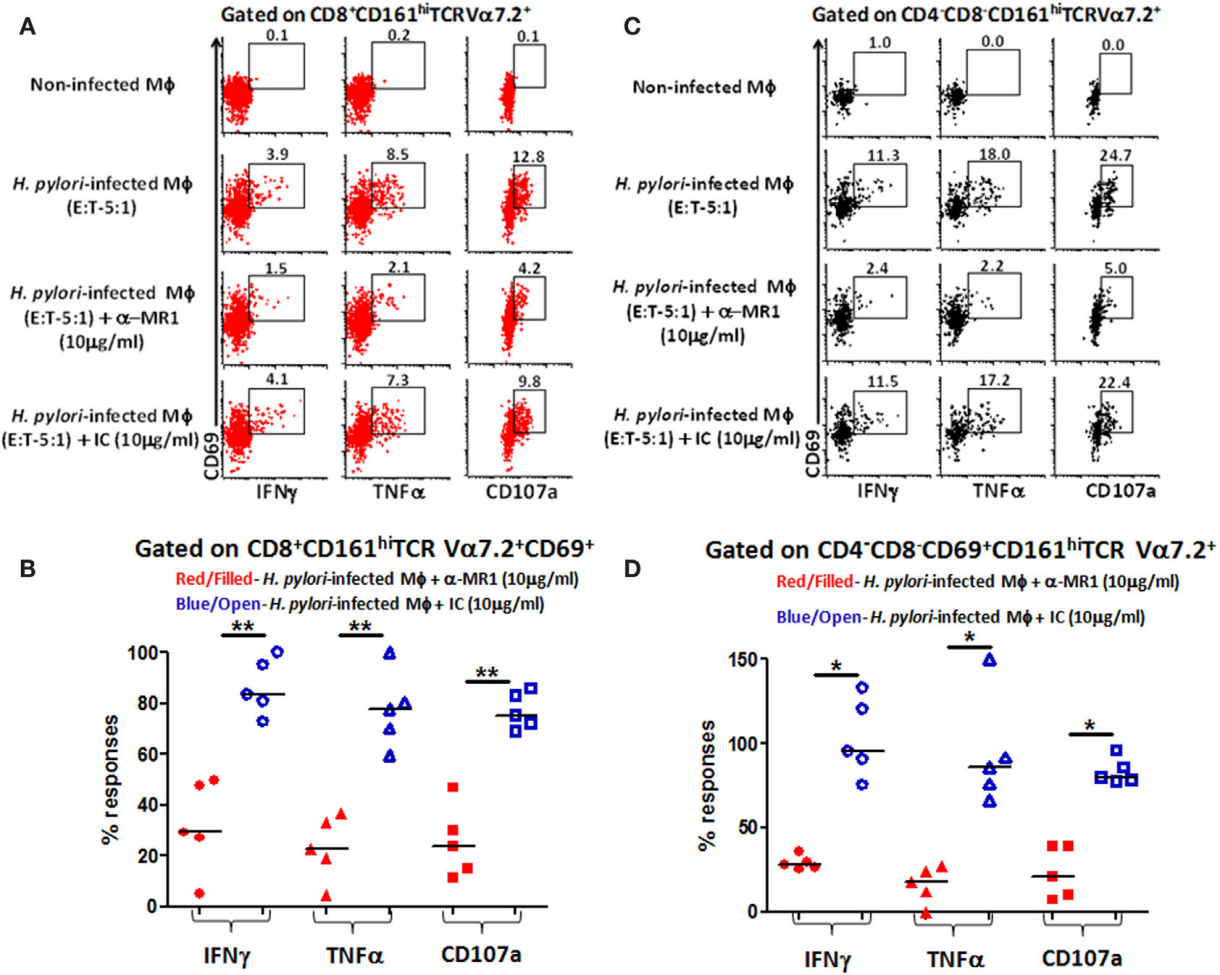
**MR1 restriction of responses by blood MAIT CD8^+^ and CD4^−^CD8^−^ DN subsets from healthy adults to *H. pylori*-infected primary autologous macrophages**. Representative volunteer showing the induction of cytokine production (IFN-γ and TNF-α) and up-regulation of CD107a expression in **(A)** CD8^+^ and **(B)** DN MAIT cell subsets following stimulation with non-infected primary autologous macrophages (Mϕ); *H. pylori*-infected primary autologous Mϕ (E:T 5:1 ratio); *H. pylori*-infected primary autologous Mϕ (E:T – 5:1 ratio) + α-MR1 (26.5 monoclonal ab) (10 μg/ml); or *H. pylori*-infected primary autologous Mϕ (E:T – 5:1 ratio) + isotype control (10 μg/ml). Cumulative data (*n* = 5) showing MR1-restricted production of IFN-γ, TNF-α, and expression of CD107a by **(C)** CD8^+^ and **(D)** DN MAIT cells following stimulation with the four treatments described above. The % of responses compared to media control (% of media control) was calculated as (cytokine production/CD107 expression in cells stimulated with *H. pylori*-infected primary autologous macrophages containing either α-MR1 or IC)/(cytokine production/CD107 expression in cells stimulated with *H. pylori*-infected primary macrophages) × 100. Horizontal black lines in **(B,D)** represent medians. Significant differences are denoted by asterisks (*P < 0.05; **P < 0.005).

Given that MAIT cells detect bacterially derived antigens presented by the MHC-like molecule MR-1, we next investigated whether responses *to H. pylori-*infected human primary autologous macrophages by CD8^+^ and DN MAIT cell subsets were MR-1 restricted. To address this question, *H. pylori*-infected primary autologous macrophages were incubated with neutralizing anti-human MR-1 antibodies or matched isotype controls to block the cytokine responses (IFN-γ and TNF-α) and/or expression of CD107a elicited in MAIT cells subsets. We observed that blocking of MR-1 using a mouse anti-human monoclonal antibody (clone 26.5; Figure 6A) resulted in significantly decreased production of cytokines (IFN-γ and TNF-α) and expression of CD107a by CD8^+^ (Figure 6A) and DN MAIT cell subsets (Figure 6B) when compared to cultures with *H. pylori*-infected autologous targets only. In contrast, the addition of a matched isotype (IC) control failed to significantly decrease the production of cytokines and expression of CD107 by CD8^+^ (Figure 6A) or DN (CD4^−^CD8^−^) (Figure 6B) MAIT cells recorded in cultures exposed to *H. pylori*-infected autologous targets. Cumulative data (*n* = 5) showed significant suppression in the production of cytokines (IFN-γ and TNF-α) and expression of CD107 by both MAIT cells subsets (CD8^+^ and DN) following blocking with MR-1 antibody but not with a matched isotype control (Figures 6C,D). These results indicate that both MAIT cell subsets detect and respond to *H. pylori*-infected primary autologous macrophages and that this effect is MR-1 restricted.

### Gastric MAIT cells activation by *H. pylori*-infected macrophages

**Figure 7:**
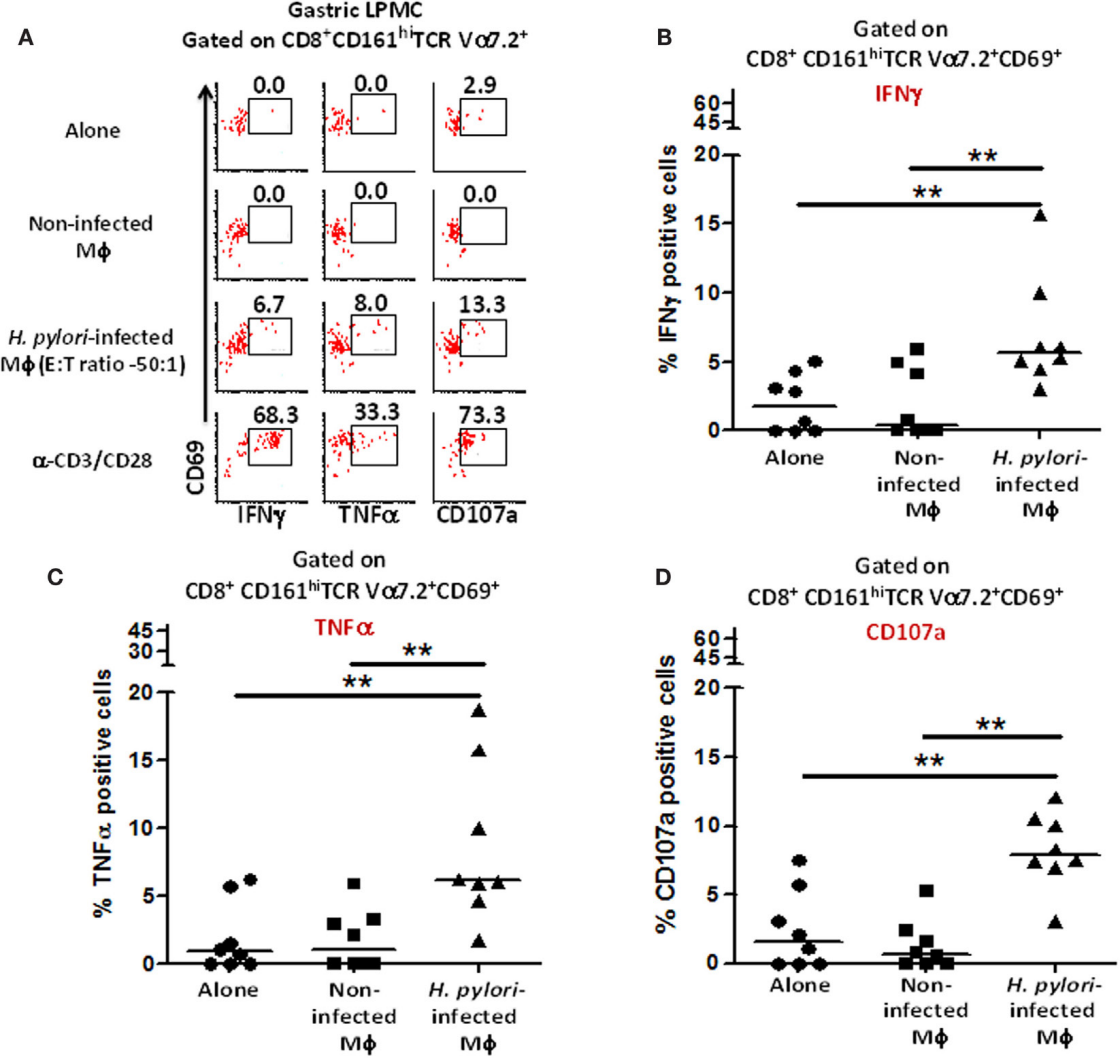
**Responses of human gastric MAIT cells to *H. pylori*-infected macrophages**. **(A)** Representative volunteer showing the induction of cytokine production (IFN-γ, TNF-α) and expression of CD107a by gastric CD8^+^ MAIT following stimulation by *H. pylori*-infected and non-infected THP-1 Mϕ. Shown are cumulative data (*n* = 8) of **(B)** IFN-γ production, **(C)** TNF-α production, and **(D)** expression of CD107a by CD8^+^ MAIT following stimulation by *H. pylori*-infected Mϕ and non-infected THP-1 Mϕ. Horizontal lines in **(B–D)** represent medians. Significant differences are denoted by **P < 0.005.

Next, we investigated whether gastric MAIT cells would recapitulate the immune responses observed in blood MAIT cells against *H. pylori*-infected THP-1 Mϕ. To address this question, isolated gastric LPMCs were stimulated with *H. pylori*-infected THP-1 Mϕ, non-infected THP-1 Mϕ, anti-CD3/CD28 beads (positive control), or media (negative control). Following stimulation with *H. pylori*-infected Mϕ, higher percentages of gastric CD8^+^ MAIT cells produced cytokines (IFN-γ and TNF-α) and up-regulated CD107a when compared to the levels following exposure to non-infected Mϕ or cells alone (cytograms from a representative subject are shown in Figure 7A). Cumulative data (*n* = 8) showed that significantly higher percentages of gastric CD8^+^ MAIT cells produced IFN-γ (5.8 ± 1.8%; mean ± SE; Figure 7B), TNF-α (8.5 ± 2.1%; mean ± SE; Figure 7C), and up-regulated CD107a (8.2 ± 1.0%; mean ± SE; Figure 7D) following stimulation with *H. pylori*-infected THP-1 Mϕ compared to non-infected Mϕ. A trend, albeit not significant, was also observed for IL-17 production (Figure 7B). We also determined the percentage of responders, i.e., volunteers who exhibited significantly increased responses (determined by *z*-tests based on the number of events collected) in the presence of gastric MAIT cells stimulated with *H. pylori*-infected THP-1 Mϕ compared to uninfected Mϕ (Figure S4 in Supplementary Material). We observed 87.5, 75, and 100% responders for IFN-γ, TNF-α, and CD107a expression, respectively (Figure S4 in Supplementary Material).

### MAIT cells are lower in PBMC of *H. pylori*-infected volunteers

**Figure 8:**
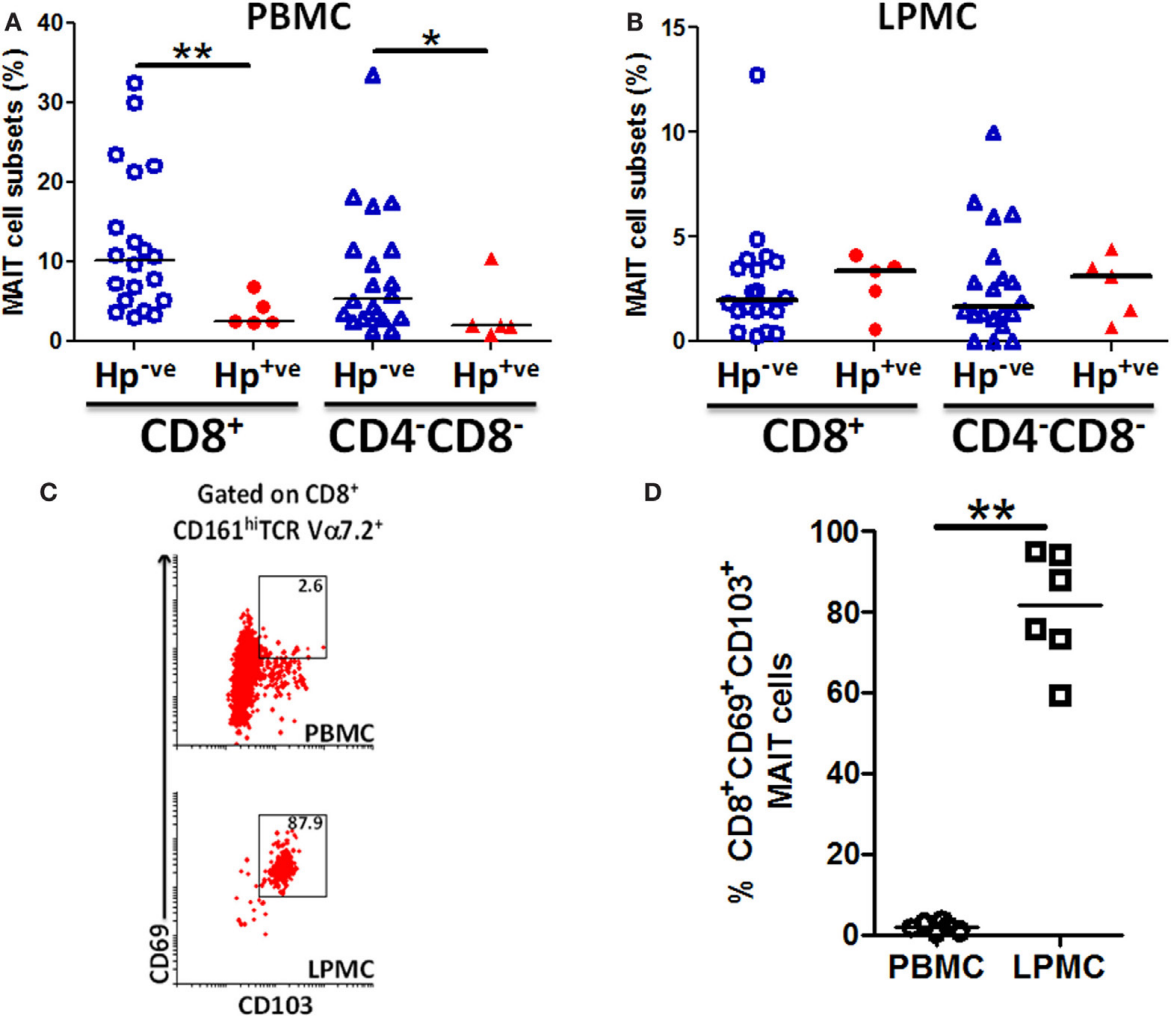
**MAIT cells are lower in blood but not in the gastric mucosa of *H. pylori*-infected volunteers**. Aggregate data of the percentages of MAIT cell subsets (CD8^+^ and CD4^−^CD8^−^) in *H. pylori*-negative (Hp^−ve^) (*n* = 20) and *H. pylori*-positive (Hp^+ve^) (*n* = 5) volunteers in **(A)** PBMC and **(B)** gastric LPMC. Hp^−ve^ volunteers were age-matched to Hp^+ve^ volunteers. **(C)** Representative example of tissue-resident T markers (CD69 and CD103) expression on CD8^+^ MAIT cells from PBMC and gastric LPMC. **(D)** Cumulative data (*n* = 6) comparing the expression of T_R_ MAIT cells in PBMC and LPMC. Horizontal lines in **(A,B,D)** represent medians. Significant differences are denoted by asterisks (*P < 0.05, **P < 0.005).

Because MAIT cells in both blood and gastric mucosa were found to be reactive to *H. pylori*-infected Mϕ, we speculated that there might be differences between MAIT cell frequencies between *H. pylori*-infected (Hp^+ve^) and uninfected (Hp^−ve^) volunteers. To explore this possibility, we assessed the frequency of MAIT cells in PBMC and gastric LPMC obtained from Hp^+ve^ and Hp^−ve^ volunteers. We observed significant decreases in the frequency of CD8^+^ MAIT cells in blood of Hp^+ve^ compared with Hp^−ve^ volunteers (Figure 8A). Analysis of the DN MAIT subsets revealed significant decreases in the frequencies of PBMC DN MAIT cells in Hp^+ve^ when compared with Hp^−ve^ volunteers (Figure 8A). These results indicated that decreased levels of CD8^+^ and DN MAIT cell subsets are present in blood during *H. pylori* infection. We then determined the frequency of MAIT cells in the gastric mucosa for the same Hp^+ve^ and Hp^−ve^ volunteers. Interestingly, no statistically significant differences were observed among the LPMC MAIT cell subset frequencies in gastric biopsies obtained from Hp^+ve^ and Hp^−ve^ groups (Figure 8B). Since our results suggest that MAIT cells in the gastric mucosa are relatively constant during aging and infection, we hypothesized that these gastric MAIT cells might represent populations of tissue-resident cells (T_R_). To explore this possibility, we assessed the hallmark markers of tissue-resident cells, i.e., expression of CD103 and CD69, on gastric LPMC and blood MAIT cells. We observed that T_R_ cells (i.e., CD103^+^ CD69^+^) represented the majority of MAIT cells in gastric LPMC, but only a small proportion in PBMC (Figures 8C,D).

## Discussion

Mucosal-associated invariant T cells are a class of innate T cells widely believed to provide immediate effector functions in response to infections in human tissues (5, 24). Here, we described, for the first time, the presence of MAIT cells in the human gastric mucosa of children, adults, and the elderly and characterized their reactivity to *H. pylori*.

Recent reports indicate a broad distribution of MAIT cells in the human body. Human MAIT cells comprise 1–10% of total T cells in peripheral blood and in lungs (13, 25), 15–39% in liver (26), and 3–5% in intestine (5). Here, we reported that the frequency of MAIT cells present in the gastric mucosa range between 0.1 and 12%. Interestingly, we observed significantly higher percentages of MAIT cells expressing a classical T_EM_ phenotype in the gastric mucosa than in peripheral blood. These results suggest that the gastric MAIT cells have the capacity to act as innate effectors for pathogens in the stomach microenvironment.

We also assessed the percentages of MAIT in peripheral blood and gastric LPMC from children, adults, and the elderly. These assessments are particularly important since few studies have compared MAIT cell frequencies among various age groups. Interestingly, we observed significantly higher percentages of MAIT cells in the blood of children when compared with those observed in adults and the elderly. Furthermore, we demonstrate that the percentages of CD8^+^ and DN MAIT subsets in peripheral blood are inversely correlated with age. These observations extend those reported in very recent studies, which showed that MAIT cell frequencies were significantly lower in PBMCs obtained from elderly volunteers than in young adult volunteers (27, 28).

These results raise an important question: what is the role of MAIT cells in children during infections (e.g., *H. pylori*)? Epidemiological studies have indicated that children infected with *H. pylori* usually develop mild *H. pylori*-associated gastritis but only rarely peptic ulcer or gastric atrophy (29, 30). Based on our observations in PBMC, it is reasonable to speculate that the higher percentages of MAIT cell in children might be associated with protection from severe disease. However, the dichotomy observed in the percentages of MAIT cells, which differ among age groups when measured in peripheral blood but not in the gastric mucosa, suggests that caution has to be exercised when interpreting data solely derived from circulating MAIT cells. The likelihood that differences exist between MAIT cells in PBMC and in the gastric mucosal is supported by our results showing that the majority of gastric MAIT cells (>80%) express tissue-resident markers (CD69^+^ CD103^+^), which are absent in PBMC MAIT. Additional studies to evaluate in detail the phenotype and function of MAIT cells in circulation and in the gastric mucosa are needed to conclusively establish the role of these MAIT cells in response to *H. pylori* infection.

The study of host immunological responses to *H. pylori* is a field of intense research (15). Following *H. pylori* colonization of the gastric mucosa, there is a significant influx of immune cells into the LP, including macrophages, dendritic cells (DCs), B cells, neutrophils, and T cells, which modulate the immunological microenvironment toward a Th1-type response (31, 32). Interestingly, these studies found an abundance of T cells in the antrum of *H. pylori*-infected donors (33). The present study is the first to describe the interactions between peripheral blood and gastric MAIT cells with *H. pylori*. We observed that MAIT from both tissues reacted to *H. pylori* by producing Th1 (TNF-α, IFN-γ) and T_H_17 (IL-17A) cytokines as well as acquiring cytotoxic effector function (expression of CD107a and lysis of *H. pylori*-infected Mϕ). Classical T cells have been implicated in the modulation of the gastric mucosa toward Th1- and Th17-type responses following *H. pylori* infection (32, 34). Here, we show that MAIT cells are also able to produce these proinflammatory cytokines following *H. pylori* stimulation. These observations suggest that MAIT cells are likely to play an important role in contributing to establish a Th1/Th17-type environment in the human gastric mucosa during *H. pylori* infection.

A key question that remains unanswered is whether MAIT cells are protective or enhance the pathogenicity of *H. pylori* infection. We hypothesize that MAIT cells may likely play an essential role in eliminating the bacteria due to its ability to produce high levels of Th1 cytokines (associated with protection) and being cytotoxic. Of note, we observed that a small fraction of MAIT cells have multifunctional capability against *H. pylori*-infected cells. These cells, which are able to produce simultaneously IFN-γ and TNF-α and exhibit cytotoxic effector functions, might be particularly effective in controlling *H. pylori* infection. In the present study, we show, using two complementary approaches, that MAIT cells have cytotoxic capabilities against *H. pylori*-infected cells. Although following *H. pylori* colonization, the bacteria usually reside in the mucus layer outside the gastric mucosa, it has been recently shown that the bacteria can infect epithelial cells and LPMC becoming a facultative intracellular bacteria (35), and a recent report has shown that MAIT cells can lyse bacterially infected epithelial cells (23). Thus, lysis of *H. pylori*-infected cells by MAIT cells could serve as a bridge between innate and classical T cells by eliminating *H. pylori*-infected cells soon after infection. Future studies directed to address MAIT cell responses to *H. pylori*-infected cells in *H. pylori*-infected and non-infected individuals, both systemically and in the gastric microenvironment, will be required to determine the precise role of MAIT cells in *H. pylori* infection.

It has recently been shown that products of the riboflavin metabolic pathway lead to MAIT cell activation. For example, 5 amino-6-d-ribitylaminouracil (5-A-RU) after non-enzymatic condensation with glyoxal or methylglyoxal results in the formation of 5-(2-oxopropylideneamino)-6-d-ribitylaminouracil (5-OP-RU), which is an unstable intermediate captured by MR-1 resulting in MAIT cell activation (36). However, it has been suggested that different bacteria might activate MAIT cells through other ligands (37). Moreover, various factors such as virulence factors, location of pathogen (e.g., intracellular), and type of APCs may also influence the response of MAIT cells to defined pathogens (38). However, since *H. pylori* express genes of the riboflavin metabolic pathway, it is likely that MAIT cells activation by *H. pylori* results, at least in part, from products of the riboflavin operon.

Another interesting observation in the present study is that the percentages of MAIT cells in PBMC of individuals infected with *H. pylori* (Hp^+ve^) are significantly decreased as compared to those from uninfected (Hp^−ve^) individuals. These results are in agreement with those observed in individuals with other active infections. For example, Le Bourhis et al. observed significantly lower proportions of MAIT cells in the blood of patients with pulmonary bacterial pathologies, including tuberculosis (4). Likewise, Gold et al. demonstrated that *M. tuberculosis* (Mtb)-reactive MAIT were decreased in PBMC but enriched in human lungs (13). These observations further support the existence of a dichotomy between MAIT cells present in peripheral blood and those in peripheral tissues. This is an important consideration since, largely due to the difficulty in obtaining peripheral tissues in humans, the interpretation of what might constitute a protective immune response is based largely on observations derived from studying peripheral blood. Our results showing decreased levels of MAIT cells in circulation suggest that these cells may have left peripheral blood to infiltrate gastric tissues after *H. pylori* infection. To address this hypothesis, we determined the percentages of MAIT cells in circulation and in the gastric mucosa of individual *H. pylori*-positive volunteers. No statistically significant differences were observed in the percentages of MAIT cells in the gastric mucosa of healthy and *H. pylori*-infected volunteers. Furthermore, we showed that the majority of gastric MAIT cells (>80%) express markers (CD69^+^ CD103^+^) compatible with tissue-resident cells, suggesting that MAIT cells can acquire a tissue-resident-like phenotype at the gastric mucosa and therefore likely to be ready to respond quickly following exposure to enteric pathogens.

Regarding the existence of multiple MAIT subsets, Gold and Lewinsohn observed that only CD8^+^ but not DN MAIT cell subsets were reactive to *M. tuberculosis* (1). Moreover, Dusseaux et al. noted that there is no evidence (phenotypic or functional) suggesting that DN or CD8 subsets are functionally distinct (12). However, to our knowledge, there are no other reports describing DN MAIT cell reactivity to pathogens. Here, we provide evidence that DN MAIT subsets are reactive to *H. pylori* and show that these responses are MR-1 restricted. In addition, our results demonstrate differences among the MAIT subsets in terms of their basal levels of activation and percentages, but not in the quality of responses, depending on whether they are in circulation or in the gastric mucosa.

In conclusion, we have demonstrated the presence of MAIT cells in the human stomach and its likely role in the host immune response to *H. pylori* infection as well as provided evidence for the role of CD8^+^ and DN MAIT cell subsets in infection.

## Author Contributions

JB performed most of the experiments, contributed to study design, acquisition of data, analysis and drafting of the manuscript; RS-G contributed to study design, analysis, and drafting of the manuscript; TB prepared *H. pylori* antigens, performed bacteria entry assays, and reviewed the manuscript; LM prepared and quantified *H. pylori* cultures and reviewed the manuscript; HK, SP, and AS performed endoscopies, obtained gastric biopsies, and reviewed the manuscript; BG performed endoscopies, obtained gastric biopsies and funding, and reviewed the manuscript; SC obtained funding and reviewed the manuscript; and MS designed the study, supervised the work, drafted the manuscript, and obtained funding.

## Conflict of Interest Statement

The authors declare that the research was conducted in the absence of any commercial or financial relationships that could be construed as a potential conflict of interest.

## Supplementary Material

The Supplementary Material for this article can be found online at http://journal.frontiersin.org/article/10.3389/fimmu.2015.00466
