# Supplementary material for: Mucosal-Associated Invariant T Cells in the Human Gastric Mucosa and Blood: Role in Helicobacter pylori Infection
Source: Front Immunol. 2015 Sep 17;6:466. doi: 10.3389/fimmu.2015.00466 (PMC4585133; doi:10.3389/fimmu.2015.00466)
Supplement: Supplementary file 1 [file Presentation_1.PDF]

Supplemental Material

Figure S1

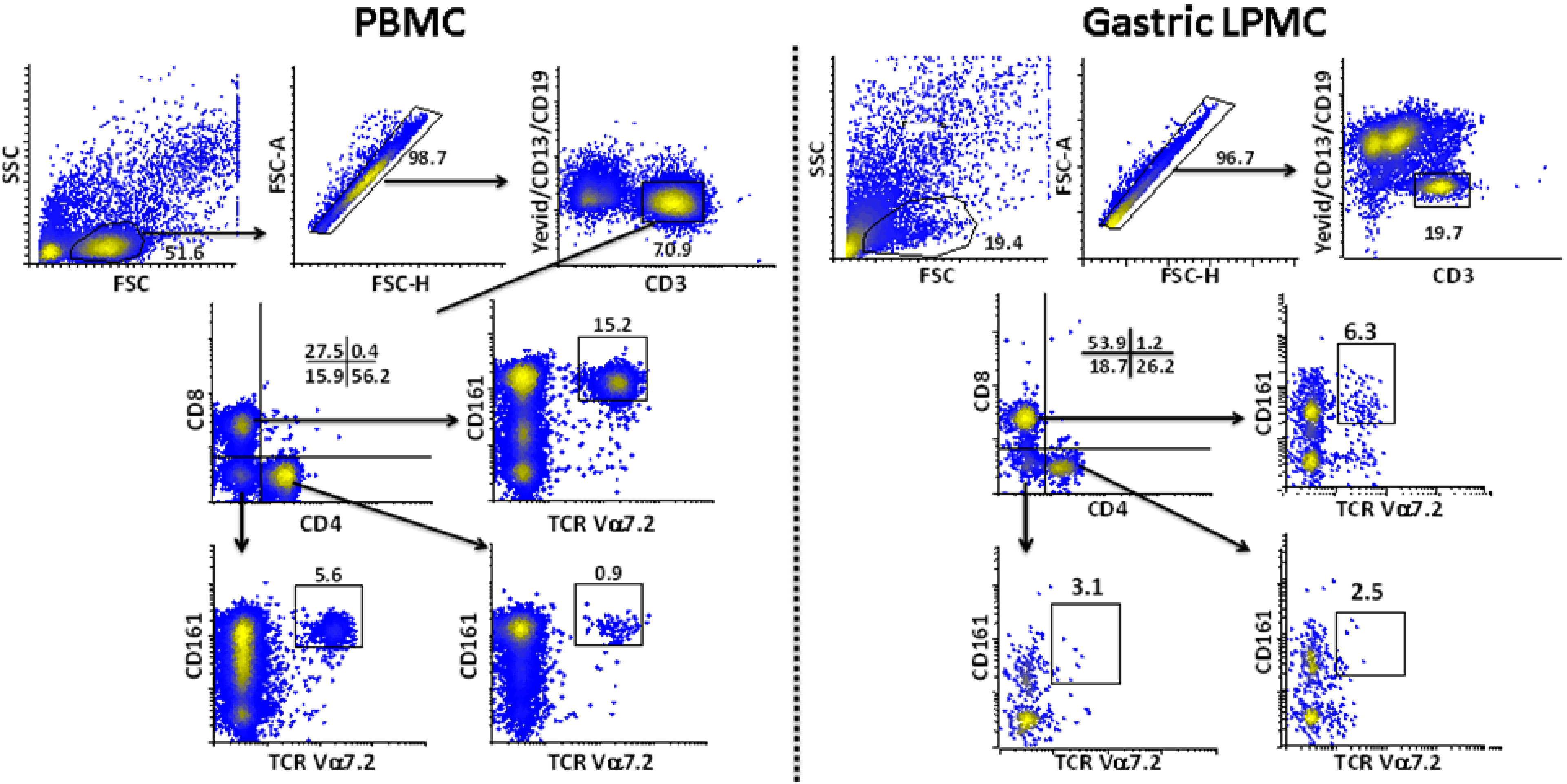

Fig S1: Complete gating strategy for identifying MAIT cells in peripheral blood and gastric tissues.

Figure S2

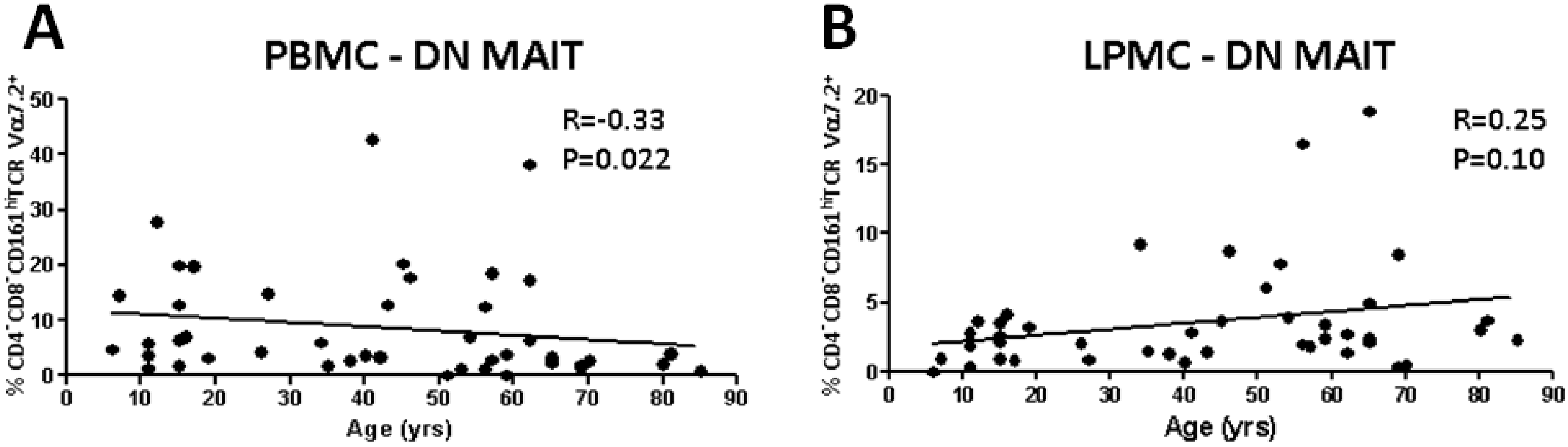

Fig S2: Correlation between the percentages of DN MAIT cells and age in peripheral blood and gastric tissues. (A) DN MAIT cells in PBMC (n=46) and **(B)** DN MAIT cells in gastric LPMC (n=43). Correlations of MAIT cells with age were performed using Spearman's correlation analysis.

Figure S3

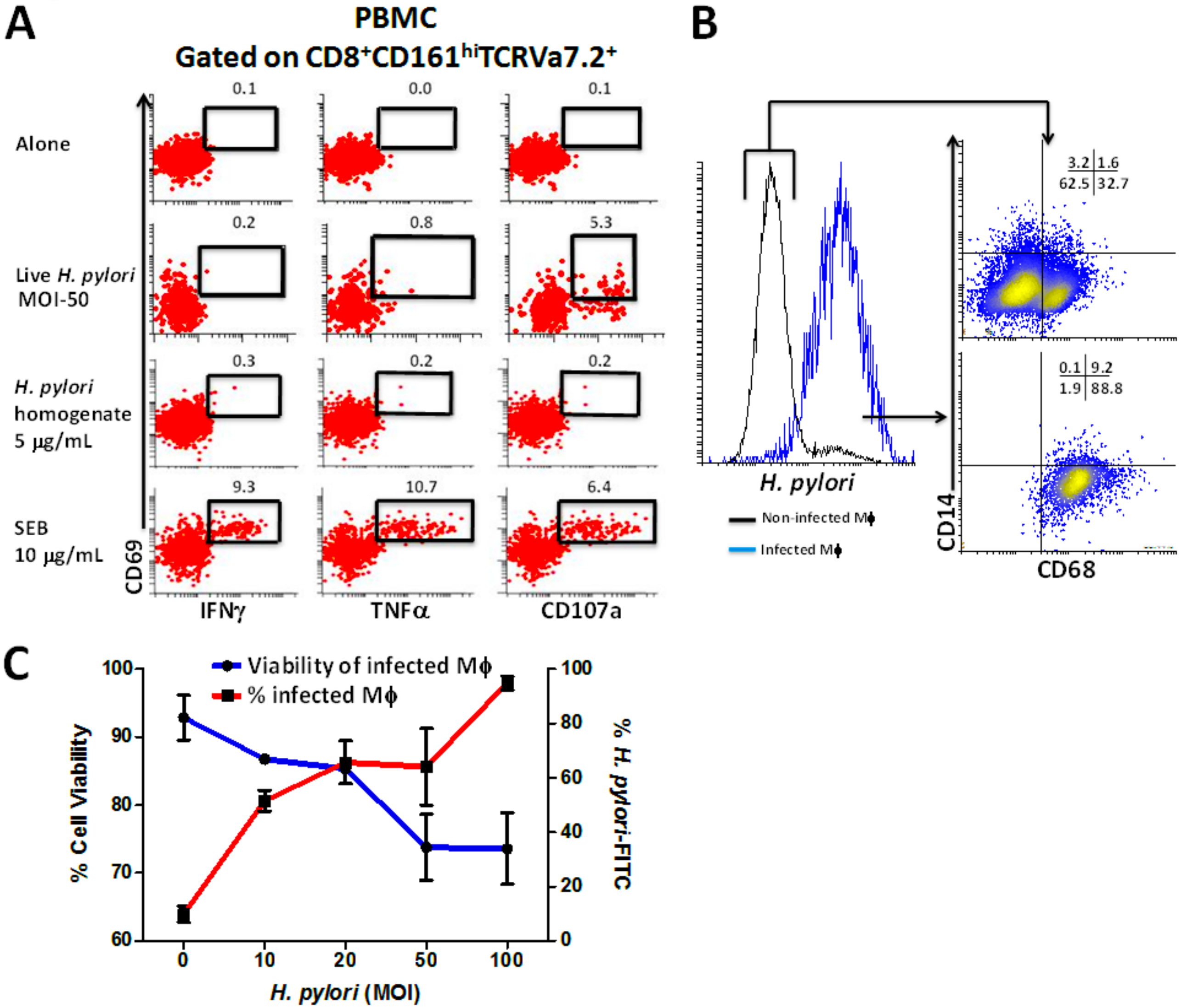

Fig S3: Blood MAIT cells exhibited moderate responses following exposure to live *H. pylori* stimulation. (A) Representative example of CD8^{+} MAIT responses (IFN-γ, TNF-α, and CD107a) following stimulation with either live *H. pylori* (MOI: 100), *H. pylori* antigens (homogenate, 5 μg/ml) or Staphylococcal enterotoxin B (SEB) (10 μg/ml). **(B)** Representative example of surface staining of *H. pylori* antigens, CD14 and CD68 on non-infected (black lines) and *H. pylori*-infected Mϕ (blue line). **(C)** THP-1 Mϕ were infected with *H. pylori* at multiple MOI (0, 10, 20, 50, 100) for 4 h at 37°C. The percentage of viable cells was determined using the yellow fluorescent live/dead reagent (blue line) and the cells harboring *H. pylori* determined by staining with an anti-*H. pylori* polyclonal antibody (red line). Data are shown as mean +/- standard error of three experiments.

Figure S4

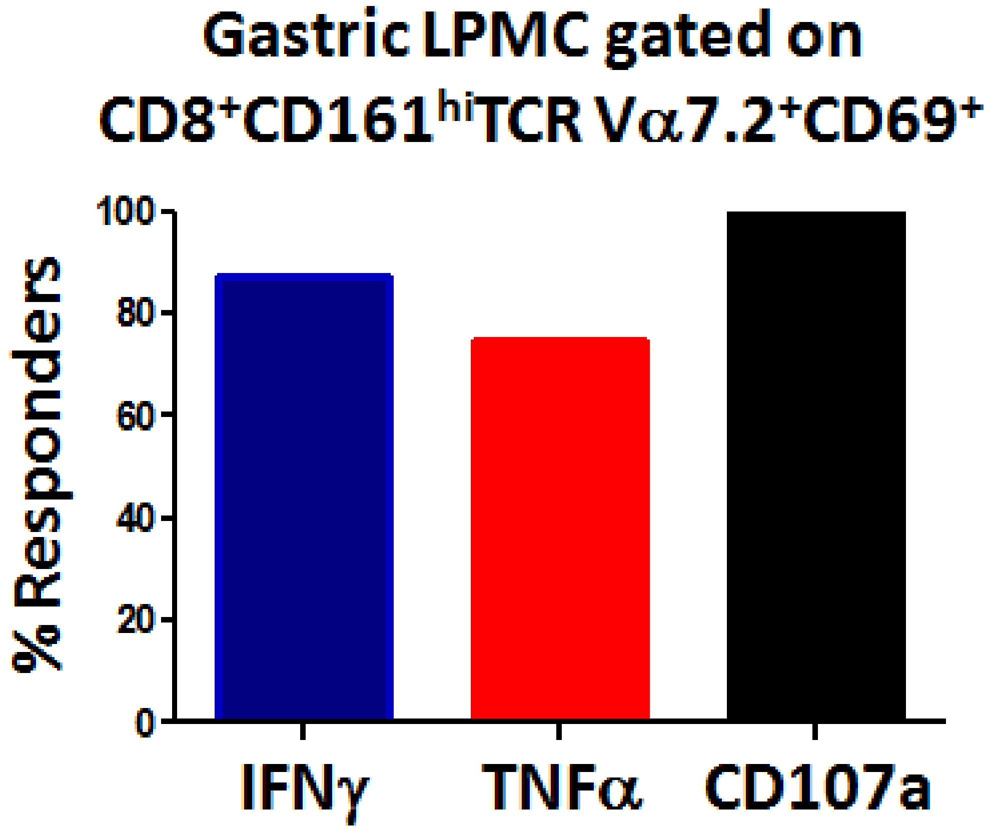

Fig S4: Percentage of responders in gastric LPMC following *H. pylori stimulation*. Responders were defined as volunteers who show significantly increased responses as determined by z-tests based on the number of positive and negative events collected in the presence of gastric LPMC stimulated with *H. pylori*-infected Mϕ compared to uninfected Mϕ. The number of responders were 7, 6 and 8 for IFNγ, TNFα, and CD107a, respectively, from the total of 8 volunteers studied.
